# Assessment of Sedative Activity of Lonicerin: In Vivo Approach With Pharmacokinetics and Molecular Docking

**DOI:** 10.1002/brb3.70524

**Published:** 2025-05-05

**Authors:** Tanzila Akter Eity, Md. Shimul Bhuia, Raihan Chowdhury, Salehin Sheikh, Siddique Akber Ansari, Nowreen Tabassum Ahammed, Hossam Kamli, Muhammad Torequl Islam

**Affiliations:** ^1^ Department of Biotechnology and Genetic Engineering Gopalganj Science and Technology University Gopalganj Bangladesh; ^2^ Department of Pharmacy Gopalganj Science and Technology University Gopalganj Bangladesh; ^3^ Bioinformatics and Drug Innovation Laboratory BioLuster Research Center Ltd. Gopalganj Bangladesh; ^4^ Department of Pharmaceutical Chemistry College of Pharmacy King Saud University Riyadh Saudi Arabia; ^5^ Department of Biology Touro University New York City New York USA; ^6^ Department of Clinical Laboratory Sciences, College of Applied Medical Sciences King Khalid University Abha Saudi Arabia; ^7^ Pharmacy Discipline Khulna University Khulna Bangladesh

**Keywords:** Insomnia, lonicerin, natural product, neuroprotective, pharmacokinetic, toxicity

## Abstract

**Background:**

Lonicerin (LON) has been identified to have different biological properties, such as anticancer, anti‐inflammatory, immunomodulatory, antibacterial, antimicrobial, and neuroprotective. This study aims to assess the sedative effect of LON in Swiss albino mice, which is yet to be discovered.

**Materials and Methods:**

Mice were treated with two different doses of LON (5 and 10 mg/kg) and 2 mg/kg of diazepam (DZP), which is the referral GABAergic medication, and the latency time and sleeping duration of animals were observed. A computational study was also conducted to evaluate the docking scores and display the binding sites of LON and receptor (GABA_A_ α1 and β2 subunits). The study also investigated the pharmacokinetics and drug‐likeness properties of LON along with toxicological analysis by using SwissADME and Protox‐3 software, respectively.

**Results:**

Findings revealed that the higher concentration of LON reduced the latency (9.86 ± 1.44 min) and increased the sleep duration (191.29 ± 7.43 min) compared to the lower concentration. Besides, the combination group of LON and DZP showed the lowest latency (6.17 ± 0.82 min) and highest sleeping time (219.00 ± 6.39 min). In the in silico study, LON exhibited a strong docking score (−8.1 kcal/mol) with the macromolecules, which is closer to the binding affinity of DZP (–8.3 kcal/mol), indicating that LON could show strong sedative activity by binding with the GABA_A_ receptor. Computational toxicity analysis revealed that LON is non‐hepatotoxic, non‐neurotoxic, noncarcinogenic, noncytotoxic, non‐ecotoxic, and non‐mutagenic.

**Conclusion:**

Therefore, LON may be effective for the treatment of insomnia in the near future.

## Graphical abstract

Lonicerin (LON) at doses of 5 and 10 mg/kg reduced sleep latency and increased sleep duration in mice, with combined LON and diazepam showing maximal effects. Computational docking showed LON binds to GABA_A_ receptor sites with a strong affinity (−8.1 kcal/mol), suggesting potential as an insomnia treatment.

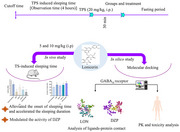

## 1 Introduction

One of the most frequent ailments in medical practice, insomnia disorder affects a substantial percentage of the population on a temporary, recurrent, or chronic basis. Dissatisfaction with the length or quality of sleep, trouble falling or staying asleep, significant distress, and impairments in daytime functioning are the primary characteristics of the disorder. It could appear as the primary symptom or, more frequently, co‐occur with other mental or physical illnesses, including depression and discomfort (Morin et al. 2015). Cognitive behavioral therapy (CBT) is usually advised as the primary intervention, but its availability is restricted by a lack of resources, which leads to the widespread use of pharmaceutical interventions as the main treatment in clinical settings (Álamo et al. 2024). The development of nonbenzodiazepine hypnotic drugs, including zolpidem, zaleplon, and eszopiclone, represented a major advancement in the treatment of insomnia. Compared to many conventional benzodiazepines, these drugs act for shorter periods and may be less likely to cause tolerance and abuse (Richey and Krystal 2011).

Gamma‐aminobutyric acid (GABA), a major inhibitory neurotransmitter in our brain, is involved in various neuronal signaling pathways that decrease the excitability of neurons and lead to a calming effect on the brain (Simeon et al. 2021). The central nervous system contains two types of GABA receptors (GABA_A_ and GABA_B_) that are involved in inhibitory synapses. The GABA_A_ receptor plays an important role in the sedation practice because different agonists of the GABA_A_ receptor target the receptor to bind and express sedative activity (Brohan and Goudra 2017). Different benzodiazepine drugs (remimazolam, 3‐hydroxyphenazapam, adinazolam, clonazolam, and deschloroetizolam) are used in the treatment of sleep disorders, which act by inducing the polysynaptic pathway inhibition by binding with GABA and altering chloride channels (Rudolph and Möhler 2006, Cornett et al. 2018). Diazepam (DZP), a common benzodiazepine, is prescribed for inducing sedation, which has side effects including sleepiness, anterograde amnesia, and confusion (which is more apparent at higher dosages). Two subunits of the GABA_A_ receptor (α1 and β2) are involved in the sedation activity (Reynolds et al. 2003). Therefore, novel therapeutics can be developed from natural products and their derivatives by focusing on these two subunits of the GABA_A_ receptor (α1 and β2).

Lonicerin (LON: C_27_H_30_O_15_), a flavonoid glycoside, is highly available in *Lonicera japonica* Thunb. They are mostly used as natural products for treating inflammatory and infectious diseases (Lv et al. 2021). It has diverse biological properties, including anticancer, anti‐inflammatory, immunomodulatory, antibacterial, antimicrobial, anti‐biofilm, antiapoptotic, and neuroprotective activities (Lv et al. 2021; Ming et al. 2022; Lee and Ma 2021; Xu et al. 2019; Gu and Sun 2020). Besides, LON could moderate different signaling pathways like NF‐κB, PI3K/Akt and MAPKs, Src/EGFR, and EZH2/NF‐κB signaling pathways (Yang et al. 2023; Park et al. 2012; Deng et al. 2022; Dai et al. 2023). Although the sedative activity of LON has yet to be discovered. Thiopental sodium (TS) is commonly used in research to evaluate sedative effects. In this model, TS is administered to animals—typically rodents—to induce sleep or sedation. Researchers then observe parameters such as the latency to sleep onset, duration of sleep, and various behavioral or physiological responses. These measurements help assess the sedative potential of TS or other test substances under investigation (Ferdous et al. 2024). This study aims to evaluate the sedative or hypnotic effect of LON through the utilization of TS‐induced sleeping time behavioral assays conducted on *Swiss* albino mice. Additionally, an in silico analysis was performed to identify potential targets responsible for the sedative or hypnotic mechanisms and to predict the pharmacokinetic (PK) and toxicological properties of LON.

## 2 Materials and Methods

### 2.1 In Vivo Study

#### 2.1.1 Chemicals and Reagents

Lonicerin (LON, CAS No. 25694‐72‐8) was kindly supplied by Chengdu Alfa Biotechnology Co. Ltd. (China), while DZP and TS were collected from Square Pharmaceuticals Ltd., Bangladesh. Tween 80 and NaCl were purchased from Merck (India).

#### 2.1.2 Experimental Animals

Adult *Mus musculus* (Swiss albino mice; avgerage b.w. 24–30 g), required for the in vivo test, were collected from the Animal House of Khulna University, Bangladesh. The animals were housed in standard conditions (temperature: 25 ± 2°C, relative humidity: 65%) for 7 days in several rectangular housing boxes (290 mm × 220 mm × 140 mm). Five mice were kept per box. Animals were kept under the free access to standard foods and water ad libitum. Studies were performed between 9:00 a.m. and 3:00 p.m. Animals involved in this experiment experienced an overnight fasting period to avoid the reaction between the components of food and the test drug. This investigation had approval from the Animal Ethics Committee of Khulna University (KUAEC‐2 023‐05‐09).

#### 2.1.3 Dose Selection and Preparation

The test doses for this study of LON (5 and 10 mg/kg) were selected from the previously published literature (Gu and Sun 2020; Lv et al. 2021). In this study, the LON dose was prepared by adding distilled water (containing 0.9% NaCl and 0.5% Tween 80). Distilled water with sodium chloride ensured isotonicity, while Tween‐80 improved the solubility and uniform dispersion of poorly water‐soluble compounds, ensuring consistent and biocompatible administration. Besides, DZP was used at a 2 mg/kg dose based on the previous literature, which found that DZP works efficiently in this concentration (Islam et al. 2024).

#### 2.1.4 In Vivo Protocols

The animals were separated into different groups, containing seven mice in each group (*n* = 7), as listed in Table 1. Besides, the vehicle (control), standard drug (DZP), and test sample (LON) were given intraperitoneally (i.p.). DZP was given at 2 mg/kg, and LON was given at 5 and 10 mg/kg, respectively. Besides, a combination of LON‐10 and DZP was given as a combination treatment. All the treatment was given i.p. After giving the treatment, each mouse was kept for 30 min. After that, 20 mg/kg b.w. of TS (i.p.) was administered to induce sleep, and then animals were observed in a plastic box. The latency time and the duration of the sleeping time of each mouse were calculated. The duration of time was observed by carefully noticing the loss and regaining of the response of animals. The sleeping time was calculated by following the physical movement or locomotion activity of mice. Animals were actually asleep, and when they moved a little bit, they were assumed to be awake, and the proper sleeping duration was calculated carefully. No movement and lying down were indicators of sleep. The provided treatments and doses are documented in Table 1.

**TABLE 1:** Different treatment groups and their doses in the sedative test.

Treatment Group	Description	Dose (mg/kg)	Target Receptor
Control (Vehicle)	Distilled water containing 0.9% NaCl and 0.5% Tween 80	10 mL/kg	—
DZP	Standard: diazepam (agonist) (i.p)	2 mg/kg	GABA_A_
LON‐5	Lower dose of lonicerin (i.p)	5 mg/kg	Under investigation
LON‐10	Higher dose of lonicerin (i.p)	10 mg/kg	Under investigation
LON‐10 + DZP	Test + Standard combination (i.p)	2 + 10 mg/kg	Under investigation

*Note*: Control (vehicle): Tween 80 and distilled water containing 0.9% NaCl and 0.5% Tween 80; DZP: Diazepam (Dose: 2 mg/kg); LON: Lonicerin (Dose: 5 and 10 mg/kg); i.p: intraperitoneally (*n* = 7).

#### 2.1.5 Statistical Analysis

The sedative effectiveness results are displayed as the mean and standard error of the mean (SEM). A one‐way analysis of variance (ANOVA) was performed, followed by the Student's *t*‐test with Tukey's multiple comparisons test, using GraphPad Prism software (version 9.5). A significance level of *p* < 0.05 was considered, with a 95% confidence interval.

### 2.2 In Silico Analysis

#### 2.2.1 Selection of the GABAA Macromolecule and Preparation

Two subunits of the GABA_A_ receptor (α1 and β2) were selected, which are related to the sedative effects according to previous literature. The 3D structures of the GABA_A_ receptor (PDB ID: 6×3X) were collected, which contains the α1 subunit in the B chain and β2 subunit in the A chain from the RCSB protein data bank, which is the only international platform that works on accumulating 3D structures of proteins and their complexes (Burley et al. 2017). After collecting the 3D structures, the macromolecule was optimized by using the software PyMOL version 1.7.4.5 to remove unnecessary fragments of amino acid and water molecules. Then, the energy consumption of the macromolecule was decreased by altering the GROMOS96 43 B1 force field by utilizing the Swiss‐PDB Viewer software program. Water, ligands, cofactors, ions, and other substances need to be eliminated in order to obtain the target's 3D coordinates from the PDB. Manipulation by editing to incorporate polar hydrogen, Kollman charge, Marge nonpolar hydrogen, and macromolecules are saved as target PDBQT, which is important to perform the molecular docking process using AutoDock Vina (Azad. 2023).

#### 2.2.2 Collection and Preparation of Ligands

**FIGURE 1:**
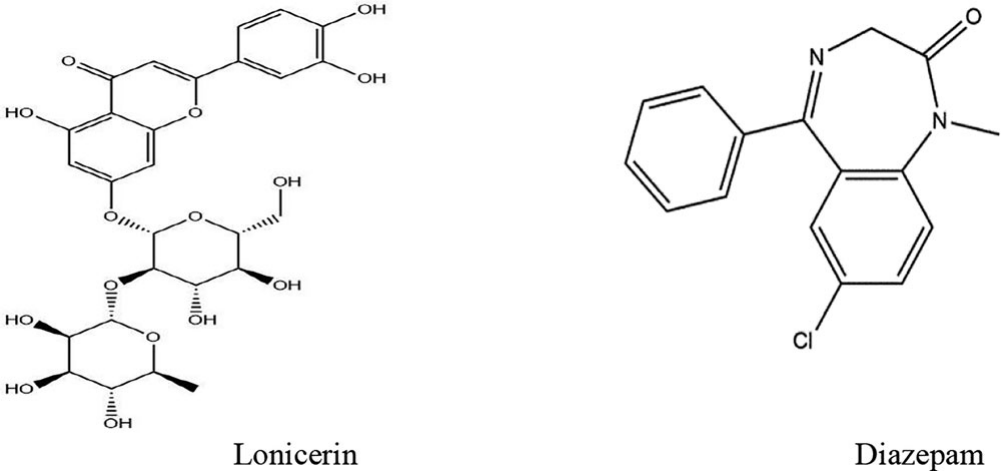
2D structure of lonicerin and diazepam.

The 3D structure of LON (PubChem ID: 5282152) and DZP (PubChem ID: 3016) was downloaded from the PubChem database in “sdf” format. After the collection, Chem3D Pro 21.0 is applied for energy reduction of the ligands by using Allinger's force field (MM2) approach. The 2D conformation of DZP and LON is presented in Figure 1.

#### 2.2.3 Docking Protocol and Non‐Bond Interaction

Molecular docking is a process that identifies the pharmacodynamic properties of the drug by analyzing the binding affinity and correlation of molecules by using PyRx v0.8 software (Harini et al. 2024). The binding scores revealed the intensity of binding affinity between the ligand and macromolecule. Besides, the results of the docking procedure also indicate the binding site of ligand and receptor (Gohlke and Klebe 2002). In this in silico study, site‐specific docking was conducted. To speed up the docking process, the size of the grid box's axes (X, Y, and Z) was adjusted to 35.02 × 39.37 × 25.00, respectively, which sped up the computation 2000 times. The PDB format of the ligand‐protein complex was created to obtain the ligand in PDBQT format. PyRx v0.8 software expressed the docking scores as a negative number. To display the binding areas of the ligand‐protein complex, BIOVIA Discovery Studio v21.1.0 was used in this investigation, which was also used to examine the non‐bond ligand interaction (Bhuia et al. 2023).

#### 2.2.4 Prediction of Drug‐Likeness and Pharmacokinetics

Pharmacokinetic (PK) characteristics help to understand and predict physiological consequences such as a substance's beneficial or detrimental impact on a particular mechanism and are crucial to the study of drugs. The SwissADME online server is used to determine the ADMET parameters and drug‐likeness properties of the drug.

#### 2.2.5 Toxicity Prediction

To identify and evaluate the compounds that have the best chance of being used safely and effectively in humans, toxicity prediction is a crucial step in the drug discovery process. Additionally, this method reduces the possibility of costly delays in the later phases of drug development (Banerjee et al. 2024). Several toxicity parameters for every given drug can be predicted using the ProTox‐3 web server. To assess the toxicity parameters, the ProTox‐3 server is required to upload the canonical SMILES, which is collected from PubChem.

## 3 Results

### 3.1 In Vivo Study

**FIGURE 2:**
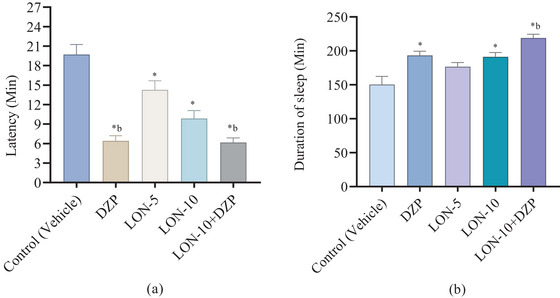
(a) latency, (b) sleeping time in minutes observed in different treatment groups of animals. (Values are mean ± SEM [*n* = 7]; one‐way ANOVA followed by *t*‐students Tukey's multiple comparisons test considering a 95% confidence interval at *p* < 0.05; *p < *0.05 compared to the *Control [vehicle], ^a^DZP, ^b^LON‐5, ^c^LON‐10, Control: Vehicle [distilled water containing 0.9% NaCl and 0.5% Tween 80]; DZP, diazepam; LON, lonicerin).

According to Figure 2, the results revealed that the referral drug DZP significantly (*p *< 0.05) decreased the latency time (6.43 ± 0.92 min) in animals compared to the control group (19.71 ± 1.81 min). In the case of LON treatment, at higher concentrations it significantly (*p *< 0.05) reduced the latency (9.86 ± 1.44 min) compared to the lower concentration (14.29 ± 1.62 min). However, the combination group of LON and DZP showed the lowest latency period (6.17 ± 0.82 min) compared to all other groups.

In terms of the calculation of duration of sleep, DZP significantly (*p *< 0.05) increased the sleeping time (193.43 ± 7.21 min) compared to the control group (150.57 ± 13.88 min). The higher concentration of LON remarkably prolonged the sleeping duration (191.29 ± 7.43 min) compared to the lower concentration (176.86 ± 6.97 min). The highest duration of sleep was calculated in the combination group of LON and DZP (219.00 ± 6.39 min).

### 3.2 In Silico Study

#### 3.2.1 Molecular Docking

**FIGURE 3:**
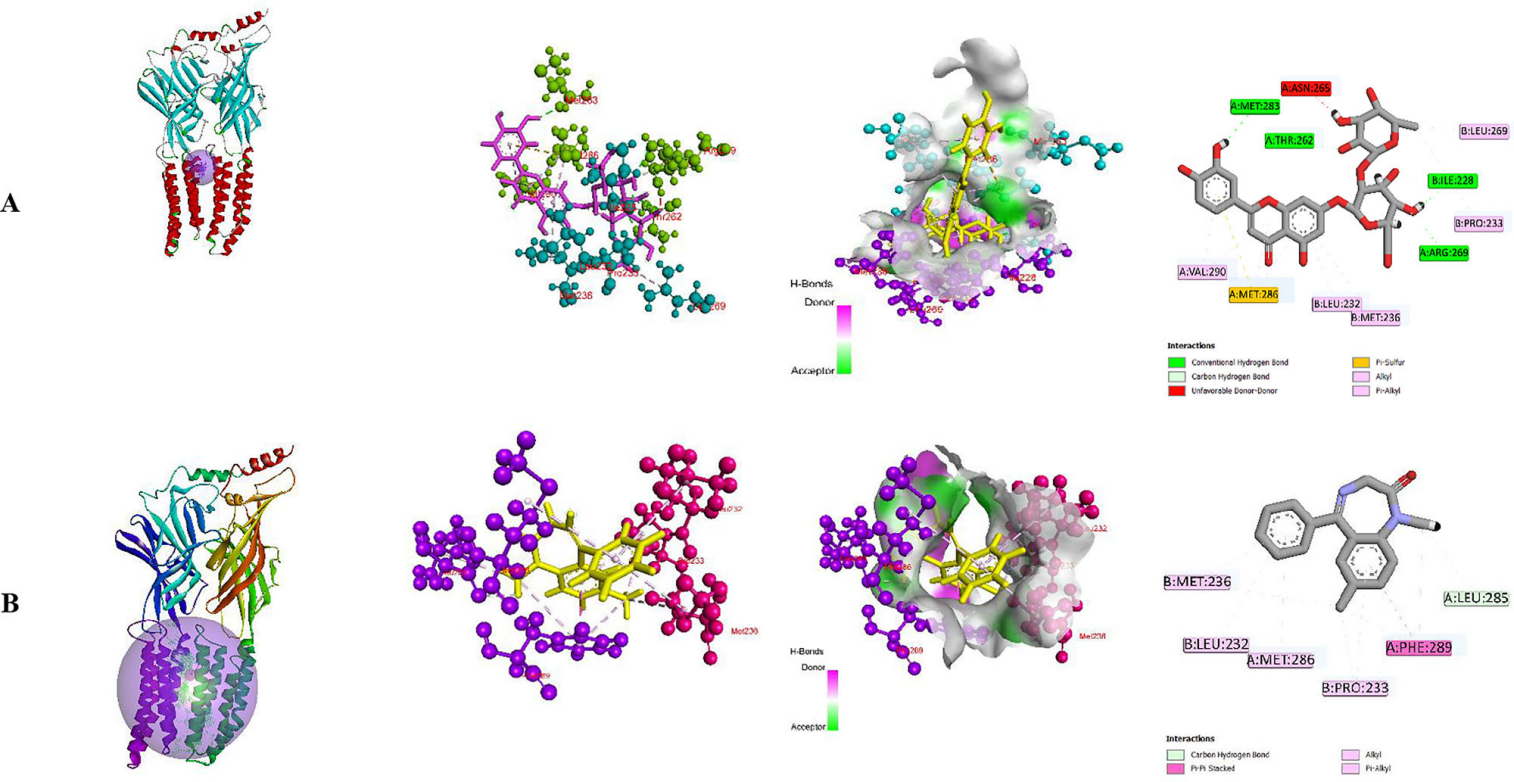
Drug‐receptor binding sites and amino acid residues of (A) Lonicerin and (B) Diazepam with GABA_A_ receptor. (DZP, diazepam; GABA, gamma‐aminobutyric acid; LON, lonicerin).

In recent years, the widely used drug developmental method is molecular docking, which estimates the degree of interaction between the macromolecule and ligand to assess the efficacy of the drug (Fan et al. 2019; Jakhar et al. 2020). In this computational study, findings demonstrated that the standard drug DZP exerted the highest binding affinity (−8.7 kcal/mol) with the α1 and β2 subunits of the GABA_A_ (6×3X) receptor. During the interaction with the macromolecules, DZP formed one hydrogen bond with the LEU285 amino acid residue and several hydrophobic bonds (π–π Stacked, Alkyl, and π‐Alkyl) with the A:PHE289, B:PRO233, A:PHE289, A:MET286, B:LEU232, and B:MET236 amino acid residues. In contrast to DZP, the test compound (LON) showed a −8.1 kcal/mol binding score, which is closer to DZP. Besides, LON interacted with the GABA_A_ receptor by forming six hydrogen bonds with B:ILE228, A:MET283, A:THR262, A:ARG269, A:THR262, and A:ARG269 amino acids with different distances and one sulfur bond (A:MET286 (π‐Sulfur)), as well as many hydrophobic bonds, including B:PRO233 (Alkyl), B:LEU269 (Alkyl), A:VAL290 (π‐Alkyl), A:MET286 (π‐Alkyl), B:LEU232 (π‐Alkyl), B:MET236 (π‐Alkyl), and A:MET286 (π‐Alkyl). The binding score, bond types, number of HBs, HB lengths, and list of AA residues liable for ligand‐receptor interactions are documented in Table 2. Figure 3 depicts both the 2D and 3D views of binding sites.

**TABLE 2:** Molecular docking scores of DZP and LON against GABA_A_ (PDB: 6×3X) receptors.

**Macromolecule**	**Ligands**	**Binding Affinity (kcal/mol)**	**No of HB**	**HB residues**	**HB distance (Å)**	**Other bonding residues**
GABA_A_	DZP	−8.7	1	A:LEU285	3.39	A:PHE289 (π–π Stacked), B:PRO233 (alkyl), B:MET236 (alkyl), A:LEU285 (alkyl), A:PHE289 (π‐alkyl), A:MET286 (π‐alkyl), B:LEU232 (π‐alkyl), B:MET236 (π‐alkyl) and B:PRO233 (π‐alkyl)
	LON	−8.1	6	B:ILE228, A:MET283, A:THR262, A:ARG269, A:THR262, A:ARG269	1.92, 2.15, 3.00, 3.23, 3.27, 3.17	A:MET286 (π‐sulfur), B:PRO233 (Alkyl), B:LEU269 (alkyl), A:VAL290 (π‐alkyl), A:MET286 (π‐alkyl), B:LEU232 (π‐alkyl), B:MET236 (π‐alkyl)

Abbreviations: DZP, diazepam; LON, lonicerin; HB, hydrogen bond.

#### 3.2.2 Drug‐Likeness and Pharmacokinetics

**FIGURE 4:**
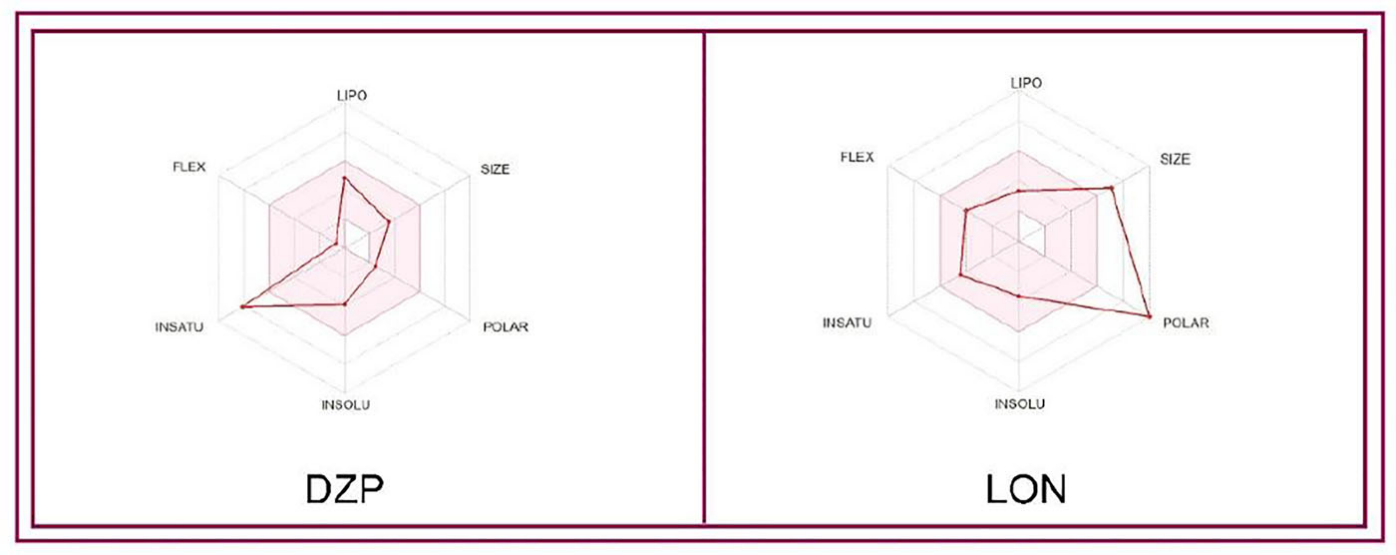
Summary of pharmacokinetic properties of diazepam and lonicerin. [The colored zone is the suitable physicochemical space for oral bioavailability; SIZE: 150 g/mol < MV < 500 g/mol; INSOLU (Insolubility): −6 < log S (ESOL) < 0; LIPO (Lipophilicity): −7 < XLOGP3 < +5.0; INSATU (In saturation): 0.25 < Fraction Csp3 < 1; POLAR (Polarity): 20 Å^2 ^< TPSA < 130 Å^2^; FLEX (Flexibility): 0 < num. Rotatable bonds < 9].

PK characteristics are essential in drug discovery since they enable the understanding and prediction of biological consequences, including the beneficial or adverse effects of a substance on a particular method (El‐Sewedy et al. 2023). In this in silico pharmacokinetics analysis, findings revealed that the molecular weight of LON and DZP is 594.52 g/mol and 284.74 g/mol, respectively. LON has a greater number of heavy atoms (42) than DZP (16). The number of hydrogen bond acceptors and donors of LON is 15 and 9, respectively. The molar refractivity (MR) of LON is 139.36, whereas the MR of DZP is 87.95. Both the DZP and LON are soluble in water. Besides, the bioavailability score of LON and DZP is 0.17 and 0.55, respectively. The skin permeation (log KP) parameter of LON was −9.67 cm/s, which indicated the accessibility of the bioactive molecule through the skin. LON showed good skin permeability, water solubility, and MR. Moreover, other PK parameters, including blood‐brain barrier (BBB) permeability, P‐gp substrate, TPSA, Log P, Log Kp, CYP1A2 inhibitor, and the CYP2C19 inhibitor score, are documented in Table 3, and a visualization is also included in Figure 4.

**TABLE 3:** Comparison of pharmacokinetic properties of diazepam and lonicerin estimated by SwissADME software.

Parameters	DZP	LON
Physicochemical Properties		
Formula	C_16_H_13_C_l_N_2O_	C_27_H_30_O_15_
Molecular weight	284.74 g/mol	594.52 g/mol
Num. heavy atoms	20	42
Num. from. heavy atoms	12	16
Fraction Csp3	0.12	0.44
Num. rotatable bonds	1	6
Num. H‐bond acceptors	2	15
Num. H‐bond donors	0	9
Molar Refractivity	87.95	139.36
TPSA	32.67 Å^2^	249.20 Å^2^
**Lipophilicity**		
Log P	2.67 2.67 2.67 2.67 2.67	−1.39
**Solubility**		
Water solubility	Soluble	Soluble
GI absorption	High	Low
BBB permeant	Yes	No
P‐gp substrate	No	Yes
CYP1A2 inhibitor	Yes	No
CYP2C19 inhibitor	Yes	No
CYP2C9 inhibitor	Yes	No
CYP2D6 inhibitor	Yes	No
CYP3A4 inhibitor	Yes	No
Log Kp (skin permeation)	−5.91 cm/s	−9.67 cm/s
**Druglikeness**		
Bioavailability Score	0.55	0.17

Abbreviations: BBB, blood‐brain barrier; GI, gastrointestinal; MLOGP, Moriguchi octanol‐water partition coefficient; PAINS, Pan‐assay interference compounds; P‐gp, P‐glycoprotein; TPSA, Topological Polar Surface Area.

#### 3.2.3 Toxicological Profile

**FIGURE 5:**
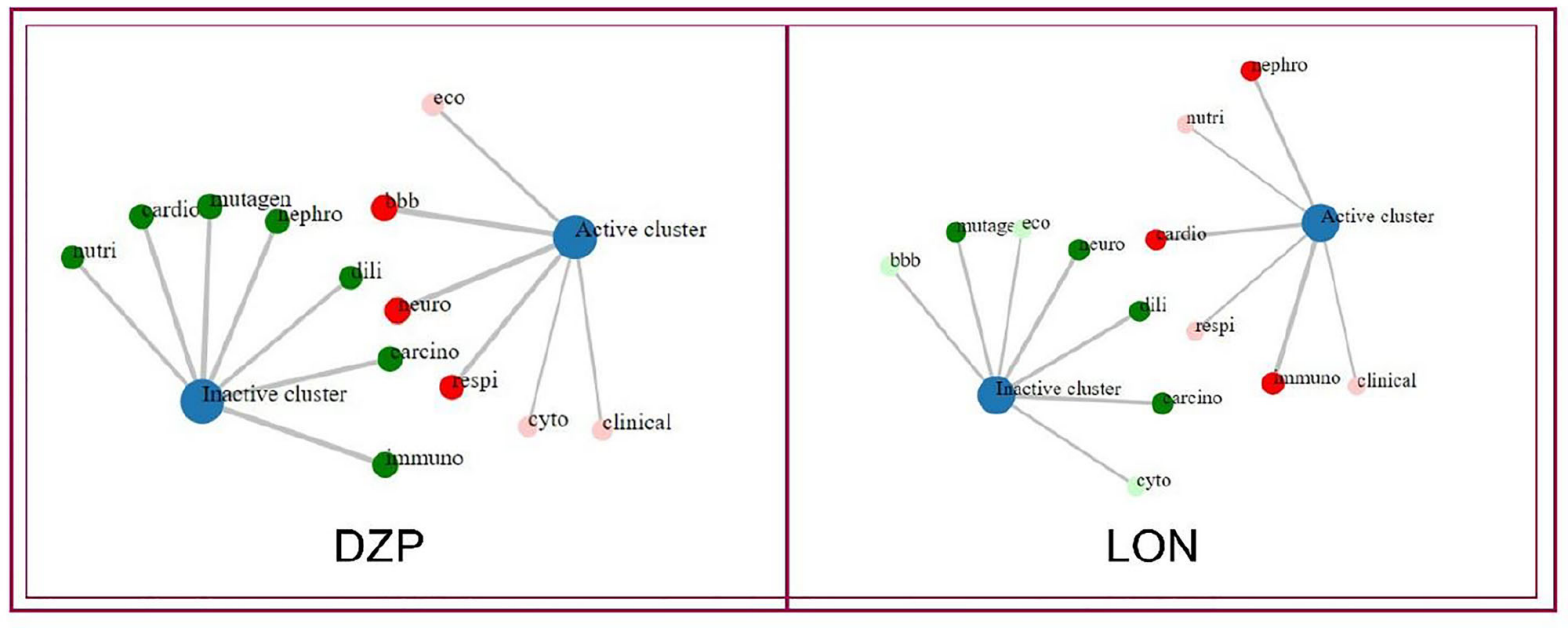
The network chart is intended to quickly illustrate the connection between the selected compound (diazepam and lonicerin) and predicted activities. (bbb, blood‐brain barrier; carcino, carcinogenicity; cardio, cardiotoxicity; clinical, clinical toxicity; cyto, cytotoxicity; dili, drug‐induced liver injury; eco, ecotoxicity; immuno, immunotoxicity; mutagen, mutagenicity; nephro, nephrotoxicity; neuro, neurotoxicity; nutri, nutritional toxicity; respi, respiratory toxicity).

In silico toxicity prediction is essential for regulating choice and lead selection in drug discovery, as in vitro and in vivo approaches are frequently constrained by ethical considerations, time, budget, and additional factors. There are different computational tools to assess the toxicity profile of any compound (Idakwo et al. 2018; Bhuia et al. 2025). In the computational toxicity analysis, results showed that the DZP is classified as Toxicity Class 2 and the predicted lethal dose (LD_50_) is 48 mg/kg, whereas LON is classified as toxicity class 5 and the LD_50_ is 5000 mg/kg, which demonstrated that the toxicity of LON is very low in high concentrations. Findings also revealed that DZP showed neurotoxicity, respiratory toxicity, cytotoxicity, ecotoxicity, and clinical toxicity but did not exhibit hepatotoxicity, nephrotoxicity, cardiotoxicity, carcinogenicity, immunotoxicity, or nutritional toxicity. On the other hand, findings also reported that LON is non‐hepatotoxic, non‐neurotoxic, noncarcinogenic, noncytotoxic, non‐ecotoxic, and non‐mutagenic. These findings confirmed the high level of safety of LON and indicated that it is a strong candidate for further research to develop as a viable drug. Findings also revealed that LON couldn't pass the BBB. All projected toxicological parameters are listed in Table 4 and also displayed in Figure 5.

**TABLE 4:** Toxicological properties of diazepam and lonicerin are estimated by Protox‐3.

Toxicity‐related parameter		DZP	LON
Organ Toxicity	Lethal Dose (LD_50_)	48 mg/kg	5000 mg/kg
	Toxicity class	2	5
	Hepatotoxicity	Inactive	Inactive
	Neurotoxicity	Active	Inactive
	Nephrotoxicity	Inactive	Active
	Respiratory toxicity	Active	Active
	Cardiotoxicity	Inactive	Active
Toxicity end points	Carcinogenicity	Inactive	Inactive
	Immunotoxicity	Inactive	Active
	Mutagenicity	Inactive	Inactive
	Cytotoxicity	Active	Inactive
	BBB‐barrier	Active	Inactive
	Ecotoxicity	Active	Inactive
	Clinical toxicity	Active	Active
	Nutritional toxicity	Inactive	Active

Abbreviations: DZP, diazepam; LON, lonicerin.

## 4 Discussion

An imbalance between excitatory and inhibitory neurotransmission in the prefrontal cortex (PFC) and associated limbic brain circuitry has been related to psychiatric disorders, most notably severe depression. Chronic stress, which also modifies excitatory and inhibitory neurotransmitter systems, often results in depression (Ghosal et al. 2017). The primary inhibitory network, the GABAergic system, is the major inhibitory system in the nervous system and is crucial for many neural functions, including neurogenesis, neuronal development, and neuroapoptosis (Henschel et al. 2008). Due to the wide range of neurotransmission activity regulated by GABA neurons, abnormalities in the GABAergic system can play a role in the pathophysiology of numerous mental illnesses, including depression (Ghosal et al. 2017). According to research, malfunctioning of GABAergic receptors may play a role in the development of depression, and depressive symptoms diminish when GABA homeostasis is restored (Fogaça and Duman 2019).

TS, a sedative or depressant, is used as a preanesthetic in operating rooms to treat several illnesses, including seizures and insomnia (Bappi et al. 2024). Through the benzodiazepine‐binding site, benzodiazepines allosterically increase the action of GABA at GABA_A_ receptors, resulting in anxiolytic, anticonvulsant, muscle‐relaxant, and sedative‐hypnotic effects (Richter et al. 2012). BZP‐like medication, such as DZP, a standard drug used for treating insomnia, induces sedation by forming a bond with the GABA_A_ receptor (Sigel and Ernst 2018). Besides, DZP stimulates the GABA_A_ receptor, which increases the chloride shift in the presynaptic neuron and leads to sedation and hypnotic effects in animals (Misra and Sharma 2020).

**FIGURE 6:**
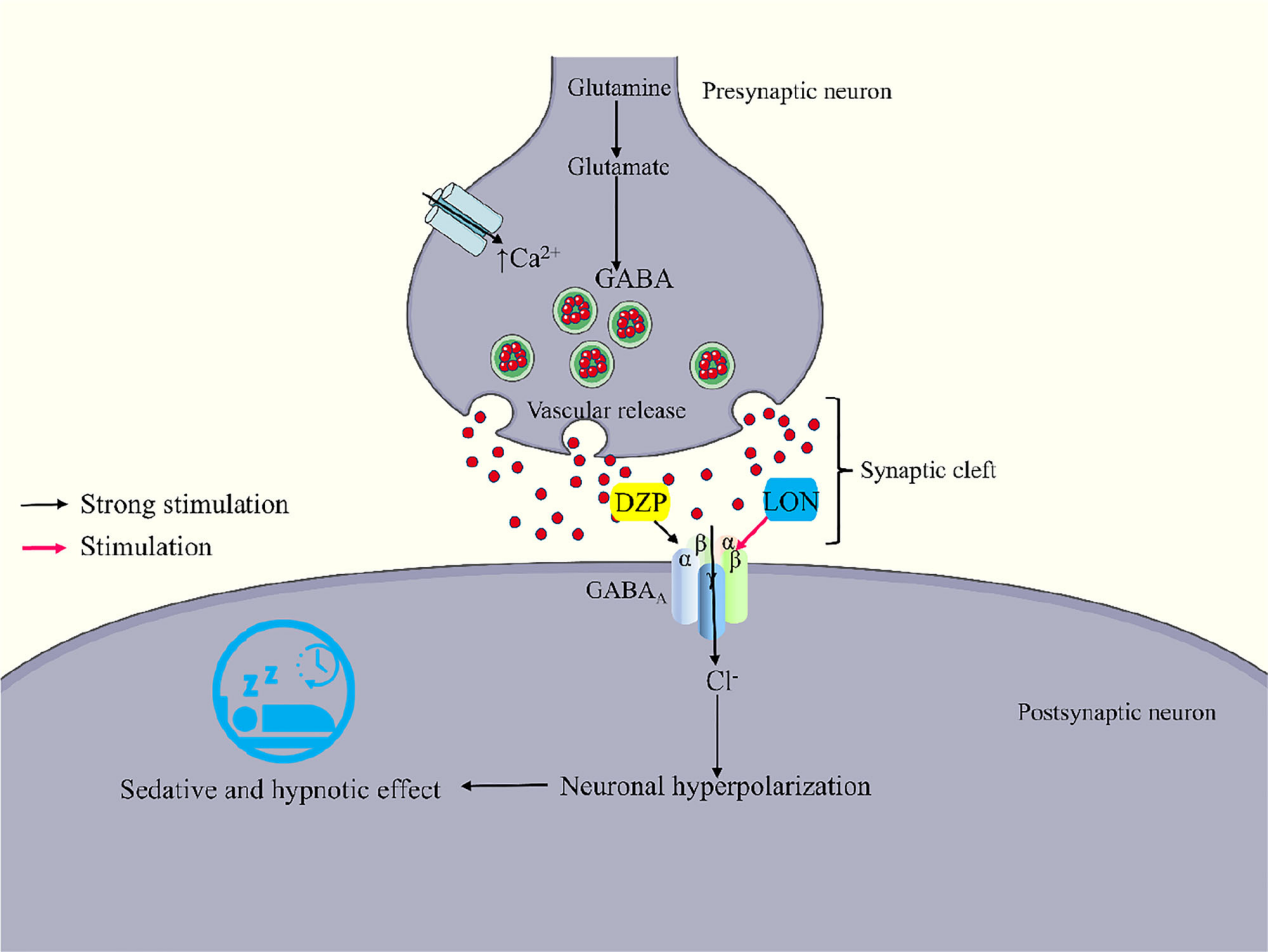
The possible sedative and hypnotic effects of LOP. The standard DZP increases the chloride ion conduction by activating the GABA_A_ receptor. (DZP, diazepam; GABA, Gamma‐aminobutyric acid; LON: lonicerin).

In the in vivo investigation, findings revealed that test compound LON significantly (*p *< 0.05) decreased latency and increased the sleep duration, where the higher concentration showed better results (Latency: 9.86 ± 1.44 min; sleeping duration: 191.29 ± 7.43 min) compared to the lower concentration (latency: 14.29 ± 1.62 min; sleeping duration: 176.86 ± 6.97 min). Besides, the combination group (LON‐10+DZP) expressed the lowest latency time (6.17 ± 0.82 min) and highest sleeping duration (219.00 ± 6.39 min) compared to the individual treatment of DZP and LON, which indicated that LON has a synergistic manner with DZP (Table 2). These findings suggested that LON could bind with the GABA_A_ receptor to exhibit DZP‐like sedation activity (Figure 6).

Molecular docking, a structure‐based drug design process, assesses the molecular interaction and evaluates the binding score and affinity between macromolecules and ligands. The field of drug design research has made extensive use of this technology in recent times (Fan et al. 2019). In the in silico study, findings revealed that the test ligand (LON) exhibited a strong binding affinity (−8.1 kcal/mol) towards the α1 and β2 subunits of the GABA_A_ receptor (PDB: 6X3X), which is closer to the binding scores of the standard drug DZP (−8.7 kcal/mol). Besides, LON formed six hydrogen bonds with the receptor (B:ILE228, A:MET283, A:THR262, A:ARG269, A:THR262, and A:ARG269), which is greater than DZP. The selectivity and effectiveness of bond formation between the ligands and receptors highly depend on the HB formation. Moreover, HB amplifies the therapeutic efficacy of the ligand (Chen et al. 2016). LON also formed several hydrophobic bonds with the receptor. Both the DZP and LON contain some amino acid residues (B:PRO233, B:MET236, A:MET286, and B:LEU232). These amino acid residues are responsible for the bond formation with the receptors, which leads to the sedation or hypnotic effect of activating the receptor.

It takes an extensive financial and time investment to bring a novel medication to market. During drug development, major bioactivities for drug candidates, such as their PKs, effectiveness, and side effects, are required to be assessed (Zhao et al. 2020). The computational PKs and drug‐likeness investigations demonstrated that LON and DZP showed MR within the range (MR ≤ 140) of 139.36 and 87.95, respectively. To evaluate the possible adverse effects of drugs, toxicology testing is required. For instance, prolonged chemical exposure in humans typically results in adverse consequences, including immune system dysfunction, genetic material damage, cancer‐causing characteristics, and harmful impacts on growth and reproduction (Raies and Bajic 2016, Genuis and Kyrillos 2017). In the computational toxicity analysis, the study found that LON did not express hepatotoxicity, neurotoxicity, carcinotoxicity, mutagenicity, cytotoxicity, and ecotoxicity. However, LON exhibited some harmful effects like nephrotoxicity, respiratory toxicity, immunotoxicity, and so forth. This study suggested further investigations into whether a combination of nano therapy to reduce these toxicity levels. The LD_50_ of LON is very high (5000 mg/kg), which indicates that LON can be used in high concentrations with less toxic effects compared to the DZP, which has a lower LD_50_ (48 mg/kg).

Taken together, LON significantly increased sleeping time in the experimental animals compared to control group. Furthermore, the in silico study revealed that LON exhibited a favorable binding affinity (−8.1 kcal/mol) toward the GABA_A_ receptor by forming multiple interactions. Therefore, LON exhibited effective sedative activity, possibly through interaction with the GABA_A_ receptor.

## 5 Conclusion

In conclusion, the findings of this study represented that LON exhibited sedative activity in TS‐induced mice. The in vivo study showed the strong sedative effects of LON‐10 by lowering the latency and increasing the sleep duration compared to the control and DZP. Besides, the combination group (LON+DZP) exhibited the lowest latency time (6.17 ± 0.82 min) and highest sleeping duration in mice (219.00 ± 6.39 min). In the case of the in silico study, LON expressed strong binding affinity (−8.1 kcal/mol) towards the α1 and β2 subunits of the GABA_A_ receptor (PDB: 6×3X), which is very close to the binding affinity of DZP (−8.7 kcal/mol) with the receptor. This study predicted the possible sedative activity of LON by binding to the GABA_A_ receptor (α1 and β2 subunits). However, several limitations of this study should be noted. Firstly, the conclusions are primarily based on behavioral tests, which may not fully capture the underlying mechanisms. Secondly, although the combination therapy increased sleeping time, the mechanism behind the additive effect on DZP activity remains unclear. Thirdly, the in silico study focused solely on the GABA_A_ receptor, which may not comprehensively explain the mechanisms of LON in the treatment of insomnia. Lastly, LON did not show BBB permeability in the in silico PK analysis, highlighting the need for validated PK studies. This limitation could potentially be overcome through nano‐based drug delivery systems or combination therapy, which may improve LON's permeability and reduce its toxicity. Further specific preclinical investigations are warranted to validate LON's safety, efficacy, potential interactions with DZP, and its therapeutic role in managing insomnia.

## Author Contributions

**Tanzila Akter Eity**: conceptualization, methodology, data curation, supervision, resources, formal analysis, software, validation, visualization, writing – review and editing, writing – original draft, investigation. **Md. Shimul Bhuia**: conceptualization, methodology, software, data curation, supervision, resources, formal analysis, writing – review and editing, visualization, validation, investigation, writing – original draft. **Raihan Chowdhury**: writing – review and editing, project administration, software, validation, writing – original draft. **Salehin Sheikh**: writing – review and editing, writing – original draft. **Siddique Akber Ansari**: validation, visualization, writing – review and editing, writing – original draft. **Nowreen Tabassum Ahammed**: resources, supervision, formal analysis, writing – original draft, visualization. **Hossam Kamli**: writing – original draft, writing – review and editing. **Muhammad Torequl Islam**: writing – review and editing, validation, visualization, writing – original draft.

## Conflicts of Interest

The authors declare no conflicts of interest.

### Peer Review

The peer review history for this article is available at https://publons.com/publon/10.1002/brb3.70524

## Data Availability

The data that support the findings of this study are available from the corresponding author upon reasonable request.

## References

[brb370524-bib-0001] Álamo, C. , J. S. Ruiz , and C. Z. Arnáez . 2024. “Orexinergic Receptor Antagonists as a New Therapeutic Target to Overcome Limitations of Current Pharmacological Treatment of Insomnia Disorder.” Actas Españolas De Psiquiatría 52, no. 2: 172. 38622003 10.62641/aep.v52i2.1659 PMC11015820

[brb370524-bib-0002] Azad, I. 2023. “Molecular Docking in the Study of Ligand‐Protein Recognition: An Overview.” In Molecular Docking‐Recent Advances, edited by E. Salih Istifli , IntechOpen.

[brb370524-bib-0039] Banerjee, P. , E. Kemmler , M. Dunkel , and R. Preissner . 2024. “ProTox 3.0: a webserver for the prediction of toxicity of chemicals.” Nucleic Acids Research 52, no. W1: W513–W520. 38647086 10.1093/nar/gkae303 PMC11223834

[brb370524-bib-0003] Bappi, M. H. , M. N. Mia , S. A. Ansari , et al. 2024. “Quercetin Increases the Antidepressant‐Like Effects of Sclareol and Antagonizes Diazepam in Thiopental Sodium‐Induced Sleeping Mice: A Possible GABAergic Transmission Intervention.” Phytotherapy Research 38, no. 5: 2198–2214. 38414297 10.1002/ptr.8139

[brb370524-bib-0004] Bhuia, M. S. , T. A. Eity , R. Chowdhury , et al. 2025. “Anxiolytic Activity of Morellic Acid: Modulation of Diazepam's Anxiolytic Effects, Possibly Through GABAergic Interventions.” CNS Neuroscience & Therapeutics 31, no. 2: e70276. 39957689 10.1111/cns.70276 PMC11831199

[brb370524-bib-0040] Bhuia, M. S. , T. Islam , M. Rokonuzzman , et al. 2023. “Modulatory effects of phytol on the antiemetic property of domperidone, possibly through the D2 receptor interaction pathway: in vivo and in silico studies.” 3 Biotech 13, no. 4: 116. 10.1007/s13205-023-03520-3 PMC10008523 36919029

[brb370524-bib-0005] Brohan, J. , and B. G. Goudra . 2017. “The Role of GABA Receptor Agonists in Anesthesia and Sedation.” CNS Drugs 31: 845–856. 29039138 10.1007/s40263-017-0463-7

[brb370524-bib-0006] Burley, S. K. , H. M. Berman , G. J. Kleywegt , J. L. Markley , H. Nakamura , and S. Velankar . 2017. “Protein Data Bank (PDB): The Single Global Macromolecular Structure Archive.” In Protein Crystallography: Methods and Protocols, edited by A. Wlodawer , Z. Dauter , and M. Jaskolski , 627–641. Humana Press. 10.1007/978-1-4939-7000-1_26 PMC5823500 28573592

[brb370524-bib-0038] Chen, D. , N. Oezguen , P. Urvil , C. Ferguson , S. M. Dann , and T. C. Savidge . 2016. “Regulation of protein‐ligand binding affinity by hydrogen bond pairing.” Science advances 2, no. 3: e1501240. 27051863 10.1126/sciadv.1501240 PMC4820369

[brb370524-bib-0007] Cornett, E. M. , M. B. Novitch , A. J. Brunk , et al. 2018. “New Benzodiazepines for Sedation.” Best Practice & Research Clinical Anaesthesiology 32, no. 2: 149–164. 30322456 10.1016/j.bpa.2018.06.007

[brb370524-bib-0008] Dai, R. , Y. Xiang , R. Fang , H. H. Zheng , Q. S. Zhao , and Y. Wang . 2023. “Lonicerin Alleviates Ovalbumin‐Induced Asthma of Mice via Inhibiting EZH2/NF‐κB Signaling Pathway.” Experimental Animals 73: 154–161. 37952975 10.1538/expanim.23-0068 PMC11091354

[brb370524-bib-0009] Deng, Z. , X. Zhang , J. Wen , et al. 2022. “Lonicerin Attenuates House Dust Mite‐Induced Eosinophilic Asthma Through Targeting Src/EGFR Signaling.” Frontiers in Pharmacology 13: 1051344. 36618942 10.3389/fphar.2022.1051344 PMC9817108

[brb370524-bib-0010] El‐Sewedy, A. , E. A. El‐Bordany , N. F. H. Mahmoud , et al. 2023. “One‐Pot Synthesis, Computational Chemical Study, Molecular Docking, Biological Study, and in Silico Prediction ADME/Pharmacokinetics Properties of 5‐Substituted 1H‐Tetrazole Derivatives.” Scientific Reports 13: 17869. 10.1038/s41598-023-44615-4. 37857636 PMC10587066

[brb370524-bib-0011] Fan, J. , A. Fu , and L. Zhang . 2019. “Progress in Molecular Docking.” Quantitative Biology 7: 83–89.

[brb370524-bib-0012] Ferdous, J. , M. S. Bhuia , R. Chowdhury , et al. 2024. “Modulatory Sedative Activity of Abrine on Diazepam in Thiopental Sodium Mediated Sleeping Mice: An In Vivo Approach With Receptor Binding Affinity of GABAergic Transmission.” ChemistrySelect 9, no. 37: e202403725. 10.1002/slct.202403725.

[brb370524-bib-0013] Fogaça, M. V. , and R. S. Duman . 2019. “Cortical GABAergic Dysfunction in Stress and Depression: New Insights for Therapeutic Interventions.” Frontiers in Cellular Neuroscience 13: 448587. 10.3389/fncel.2019.00087 PMC6422907 30914923

[brb370524-bib-0014] Genuis, S. J. , and E. Kyrillos . 2017. “The Chemical Disruption of Human Metabolism.” Toxicology Mechanisms and Methods 27, no. 7: 477–500. 28446067 10.1080/15376516.2017.1323986

[brb370524-bib-0015] Ghosal, S. , B. D. Hare , and R. S. Duman . 2017. “Prefrontal Cortex GABAergic Deficits and Circuit Dysfunction in the Pathophysiology and Treatment of Chronic Stress and Depression.” Current Opinion in Behavioral Sciences 14: 1–8. 27812532 10.1016/j.cobeha.2016.09.012 PMC5086803

[brb370524-bib-0016] Gohlke, H. , and G. Klebe . 2002. “Approaches to the Description and Prediction of the Binding Affinity of Small‐Molecule Ligands to Macromolecular Receptors.” Angewandte Chemie International Edition 41, no. 15: 2644–2676. 12203463 10.1002/1521-3773(20020802)41:15<2644::AID-ANIE2644>3.0.CO;2-O

[brb370524-bib-0017] Gu, L. Z. , and H. Sun . 2020. “Lonicerin Prevents Inflammation and Apoptosis in LPS‐induced Acute Lung Injury.” Frontiers in Bioscience‐Landmark 25, no. 3: 480–497. 10.2741/4815 31585898

[brb370524-bib-0018] Harini, M. , K. Kavitha , V. Prabakaran , et al. 2024. “Identification of Apigenin‐4ʹ‐Glucoside as Bacterial DNA Gyrase Inhibitor by QSAR Modeling, Molecular Docking, DFT, Molecular Dynamics, and In Vitro Confirmation Studies.” Journal of Molecular Modeling 30, no. 1: 22. 38170229 10.1007/s00894-023-05813-z

[brb370524-bib-0019] Henschel, O. , K. E. Gipson , and A. Bordey . 2008. “GABAA Receptors, Anesthetics and Anticonvulsants in Brain Development.” CNS & Neurological Disorders‐Drug Targets (Formerly Current Drug Targets‐CNS & Neurological Disorders) 7, no. 2: 211–224. 10.2174/187152708784083812 PMC2557552 18537647

[brb370524-bib-0020] Idakwo, G. , J. Luttrell , M. Chen, et al. 2018. “A Review on Machine Learning Methods for In Silico Toxicity Prediction.” Journal of Environmental Science and Health, Part C 36, no. 4: 169–191. 10.1080/10590501.2018.1537118 30628866

[brb370524-bib-0021] Islam, M. T. , J. Ferdous , M. S. Al Hasan , et al. 2024. “Phytol Exerts Sedative‐Like Effects and Modulates the Diazepam and Flumazenil's Action, Possibly Through the GABAA Receptor Interaction Pathway.” Neuroscience Letters 842: 138007. 39357640 10.1016/j.neulet.2024.138007

[brb370524-bib-0022] Jakhar, R. , M. Dangi , A. Khichi , and A. K. Chhillar . 2020. “Relevance of Molecular Docking Studies in Drug Designing.” Current Bioinformatics 15, no. 4: 270–278.

[brb370524-bib-0023] Lee, H. , and C. J. Ma . 2021. “Neuroprotective Activity of Lonicerin Isolated From *Lonicera japonica* .” Korean Journal of Pharmacognosy 52, no. 1: 19–25.

[brb370524-bib-0024] Lv, Q. , Y. Xing , J. Liu , et al. 2021. “Lonicerin Targets EZH2 to Alleviate Ulcerative Colitis by Autophagy‐Mediated NLRP3 Inflammasome Inactivation.” Acta Pharmaceutica Sinica B 11, no. 9: 2880–2899. 34589402 10.1016/j.apsb.2021.03.011 PMC8463273

[brb370524-bib-0025] Ming, X. , M. Yin , and W. Liyan . 2022. “Antibacterial and Anti‐Inflammatory Potential of Chinese Medicinal Herbs: *Lonicerae flos*, *Lonicerae japonicae flos*, *Scutellaria baicalensis* Georgi, and *Forsythia suspensa* .” Natural Product Communications 17, no. 11: 1934578×221136673.

[brb370524-bib-0026] Misra, A. K. , and P. K. Sharma . 2020. “Sedative and Hypnotic Drugs.” In Advances in Neuropharmacology, edited by M. S. Uddin and M. Rashid , 259–282. Apple Academic Press.

[brb370524-bib-0027] Morin, C. M. , C. L. Drake , A. G. Harvey , et al. 2015. “Insomnia Disorder.” Nature Reviews Disease Primers 1, no. 1: 15026. 10.1038/nrdp.2015.26 27189779

[brb370524-bib-0028] Richey, S. M. , and A. D. Krystal . 2011. “Pharmacological Advances in the Treatment of Insomnia.” Current Pharmaceutical Design 17, no. 15: 1471–1475. 21476952 10.2174/138161211796197052

[brb370524-bib-0029] Park, H. S. , K. I. Park , D. H. Lee , et al. 2012. “Polyphenolic Extract Isolated From Korean *Lonicera japonica* Thunb. Induce G2/M Cell Cycle Arrest and Apoptosis in HepG2 Cells: Involvements of PI3K/Akt and MAPKs.” Food and Chemical Toxicology 50, no. 7: 2407–2416. 22561682 10.1016/j.fct.2012.04.034

[brb370524-bib-0030] Raies, A. B. , and V. B. Bajic . 2016. “In Silico Toxicology: Computational Methods for the Prediction of Chemical Toxicity.” Wiley Interdisciplinary Reviews: Computational Molecular Science 6, no. 2: 147–172. 27066112 10.1002/wcms.1240 PMC4785608

[brb370524-bib-0041] Reynolds, D. S. , T. W. Rosahl , J. Cirone , et al. 2003. “Sedation and anesthesia mediated by distinct GABA(A) receptor isoforms.” The Journal of neuroscience: The official journal of the Society for Neuroscience 23, no. 24: 8608–8617. 13679430 10.1523/JNEUROSCI.23-24-08608.2003 PMC6740367

[brb370524-bib-0031] Richter, L. , C. De Graaf , W. Sieghart , et al. 2012. “Diazepam‐Bound GABAA Receptor Models Identify New Benzodiazepine Binding‐Site Ligands.” Nature Chemical Biology 8, no. 5: 455–464. 22446838 10.1038/nchembio.917 PMC3368153

[brb370524-bib-0032] Rudolph, U. , and H. Möhler . 2006. “GABA‐Based Therapeutic Approaches: GABAA Receptor Subtype Functions.” Current Opinion in Pharmacology 6, no. 1: 18–23. 16376150 10.1016/j.coph.2005.10.003

[brb370524-bib-0033] Sigel, E. , and M. Ernst . 2018. “The Benzodiazepine Binding Sites of GABAA Receptors.” Trends in Pharmacological Sciences 39, no. 7: 659–671. 29716746 10.1016/j.tips.2018.03.006

[brb370524-bib-0034] Simeon, J. O. , J. O. Simeon , S. A. Zubairu , and A. D. Adegbenga . 2021. “Concomitant Administration of Ethanol Leaf Extract of *Thymus vulgaris* on Diazepam‐Induced Sedation and Hypnosis in Wister Rat.” Journal of Nursing and Health Science 16, no. 5: 04–09.

[brb370524-bib-0035] Xu, Z. , K. Li , T. Pan , et al. 2019. “Lonicerin, an Anti‐algE Flavonoid Against *Pseudomonas aeruginosa* Virulence Screened From Shuanghuanglian Formula by Molecule Docking Based Strategy.” Journal of Ethnopharmacology 239: 111909. 31026553 10.1016/j.jep.2019.111909

[brb370524-bib-0036] Yang, X. , H. Qian , J. Meng , et al. 2023. “Lonicerin Alleviates the Progression of Experimental Rheumatoid Arthritis by Downregulating M1 Macrophages Through the NF‐κB Signaling Pathway.” Phytotherapy Research 37, no. 9: 3939–3950. 37114508 10.1002/ptr.7853

[brb370524-bib-0037] Zhao, L. , H. L. Ciallella , L. M. Aleksunes , and H. Zhu . 2020. “Advancing Computer‐Aided Drug Discovery (CADD) by Big Data and Data‐Driven Machine Learning Modeling.” Drug Discovery Today 25, no. 9: 1624–1638. 32663517 10.1016/j.drudis.2020.07.005 PMC7572559

